# Unambiguous evidence of brilliant iridescent feather color from hollow melanosomes in an Early Cretaceous bird

**DOI:** 10.1093/nsr/nwab227

**Published:** 2021-12-28

**Authors:** Yanhong Pan, Zhiheng Li, Min Wang, Tao Zhao, Xiaoli Wang, Xiaoting Zheng

**Affiliations:** State Key Laboratory for Mineral Deposits Research, School of Earth Sciences and Engineering, Centre for Research and Education on Biological Evolution and Environment and Frontiers Science Center for Critical Earth Material Cycling, Nanjing University, China; Key Laboratory of Vertebrate Evolution and Human Origins of Chinese Academy of Sciences, Institute of Vertebrate Paleontology and Paleoanthropology, Chinese Academy of Sciences, China; CAS Center for Excellence in Life and Paleoenvironment, China; Key Laboratory of Vertebrate Evolution and Human Origins of Chinese Academy of Sciences, Institute of Vertebrate Paleontology and Paleoanthropology, Chinese Academy of Sciences, China; CAS Center for Excellence in Life and Paleoenvironment, China; State Key Laboratory for Mineral Deposits Research, School of Earth Sciences and Engineering, Centre for Research and Education on Biological Evolution and Environment and Frontiers Science Center for Critical Earth Material Cycling, Nanjing University, China; Institute of Geology and Paleontology, Linyi University, China; Shandong Tianyu Museum of Nature, China; Institute of Geology and Paleontology, Linyi University, China; Shandong Tianyu Museum of Nature, China

## Abstract

A unique form of melanosomes contributing to brilliant iridescent colors in modern bird feathers, previously unknown in fossil birds, is identified in the Early Cretaceous bird Eoconfuciusornis. The discovery highlights the complexity of plumage color nanostructures utilized early in bird evolution as far back as 130 million years ago.

Various plumage colors are roughly produced by pigment-based colors and structural colors [1,2]. Brilliant iridescent plumage colors (in which the color changes depending on the reflectance spectra of sunlight at different viewing angles) greatly expand the range of plumage colors and create colors unachievable by plants or other animals [1]. Iridescence is produced by light interference with photonic crystals, which have periodic changes in their refractive index (RI). Iridescent plumage color is produced by coherent light scattering from periodic ordered stacks of melanosomes within keratin. Based on extensive studies of the melanosomes in iridescent feathers in extant birds, melanosomes that create iridescent plumage colors are broadly classified into four types: solid cylindrical, solid flattened, hollow cylindrical and hollow flattened [2–4]. The flat and hollow forms are found exclusively in iridescent feathers [4].

Studies of fossil melanosomes give us a unique opportunity to explore potential patterns of melanosome diversification, and help to reconstruct the evolution of avian plumage color. Fossil melanosomes were first detected in preserved feathers more than a decade ago [5], which has enabled structural color to be detected in extinct animals based on the melanosome morphology and organized arrays of melanosomes in regular and nanoscale patterns (e.g. [6]).

Here, we further studied the nanostructures of the melanosome sampled from a *Eoconfuciusornis* specimen with Scanning Electron Microscope (SEM) and Transmission Electron Microscope (TEM) analyses (for details on materials and methods see Supplementary Data). The fossil melanosomes are clearly visible as hollow rods with air holes that are roughly circular in cross section (Fig. 1, Fig. S1); however, due to taphonomic alterations some melanosomes are more or less fused, resulting in the merged appearance of air holes (Fig. 1B; also see Supplementary Data). In most cases the cores of individual melanosomes have lower electron density than the exterior. They appear to be randomly oriented, with rodlets' cross sections ranging from oval to ellipse (Fig. 1C). The observed nanostructures strongly resemble the hollow melanosome type present in the iridescent feathers of some modern birds, such as African starlings, birds of paradise and wild turkeys [7], representing the oldest record of avian hollow melanosomes (Fig. 1).

**Figure 1.**
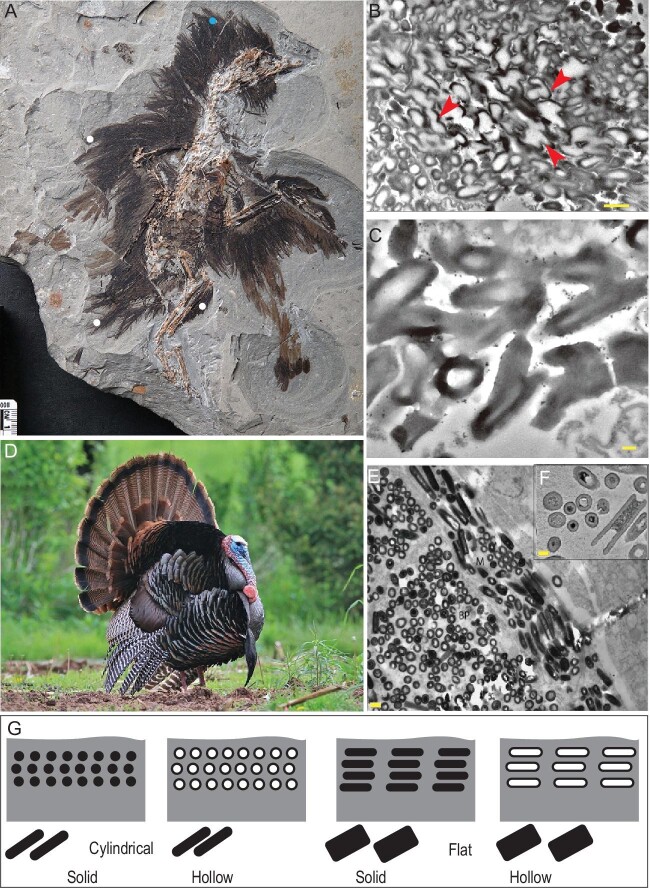
Melanosomes from *Eoconfuciusornis* (STM 7-144) compared with the modern wild turkey. (A) Dots indicate the sample locations for TEM analyses, and the hollow melanosome is collected from the blue colored one. (B and C) TEM images of cross section melanosomes from *Eoconfuciusornis*; the scale bar in (B) is 1000 nm, and that in (C) is 200 nm. (D) A male wild turkey, *Meleagris gallopavo*. (E and F) TEM images of cross section melanosomes from *M. gallopavo*, adapted from Ref. [7]. Scale bar in (E) is 1000 nm, and that in (F) is 200 nm. (G) Schematic drawings of the four main types of melanosomes found in modern iridescent feathers. Red arrows in (B) indicate merged air holes due to fusion of melanosomes.

Compared to solid melanosomes containing melanin with RI = ∼2.0 and β-keratin with RI = ∼1.58, hollow melanosomes introduce air with extremely low RI = ∼1.0, and therefore produce brighter colors owing to a higher RI contrast than the solid melanosomes [3]. It has been shown that birds with more complex melanosomes and variable melanosome types forming the photonic crystals could increase the range of color variability [2,4], e.g. the colorful hummingbirds that contain the most complicated hollow and flattened melanosomes [2]. In modern birds, modification of melanosome morphologies causing additional interfaces between high and low RI materials can further enrich colorations that are associated with radiations on some well-known avian clades, for example, hummingbirds, birds of paradise, sunbirds and trogons [2]. Further studies demonstrate that the evolution of optically innovative melanosomes positively affects the accumulation of morphological disparity and lineage diversification [2].

It is probable that the hollow melanosomes in *Eoconfuciusornis* evolved independently of those in crown birds. In *Eoconfuciusornis*, hollow melanosomes were found on the top of the head, which is consistent with the interpretation that coloration could be additional evidence of ornamentation, in addition to the tail feathers. There is mounting evidence that the feathers or feather-like structures discovered in feathered-dinosaurs (including birds) exhibit numerous morphotypes relating to sexual display (e.g. [8] and references in it), for example, the paired, elongated tail feathers in *Eoconfuciusornis* and *Confuciusornis*, the pennaceous rectrices in *Caudipteryx*, the ribbon-like tail feathers in *Epidexipteryx* and the rectricial fan combined with elongated central paired pennaceous plumes in *Yuanchuornis*. The available fossil evidence indicates that birds had diversified globally by the Early Cretaceous [9]. In addition to the appearance of skeletal and other biological innovations [10], the occurrence of hollow melanosomes in early branching avialans such as *Eoconfuciusornis* may have also contributed to evolution through sexual selection, with enhanced visual signals afforded by diverse plumage colors.

## Supplementary Material

nwab227_Supplemental_File
