# Supplementary material for: Unambiguous evidence of brilliant iridescent feather color from hollow melanosomes in an Early Cretaceous bird
Source: Natl Sci Rev. 2021 Dec 28;9(2):nwab227. doi: 10.1093/nsr/nwab227 (PMC8824705; doi:10.1093/nsr/nwab227)
Supplement: nwab227_Supplemental_File [file nwab227_supplemental_file.docx]

**Supplementary Data for**

**Unambiguous evidence of brilliant iridescent feather color from hollow melanosomes in an Early Cretaceous bird**

including

Supporting Figures S1-S4

Supplementary Texts 1-2


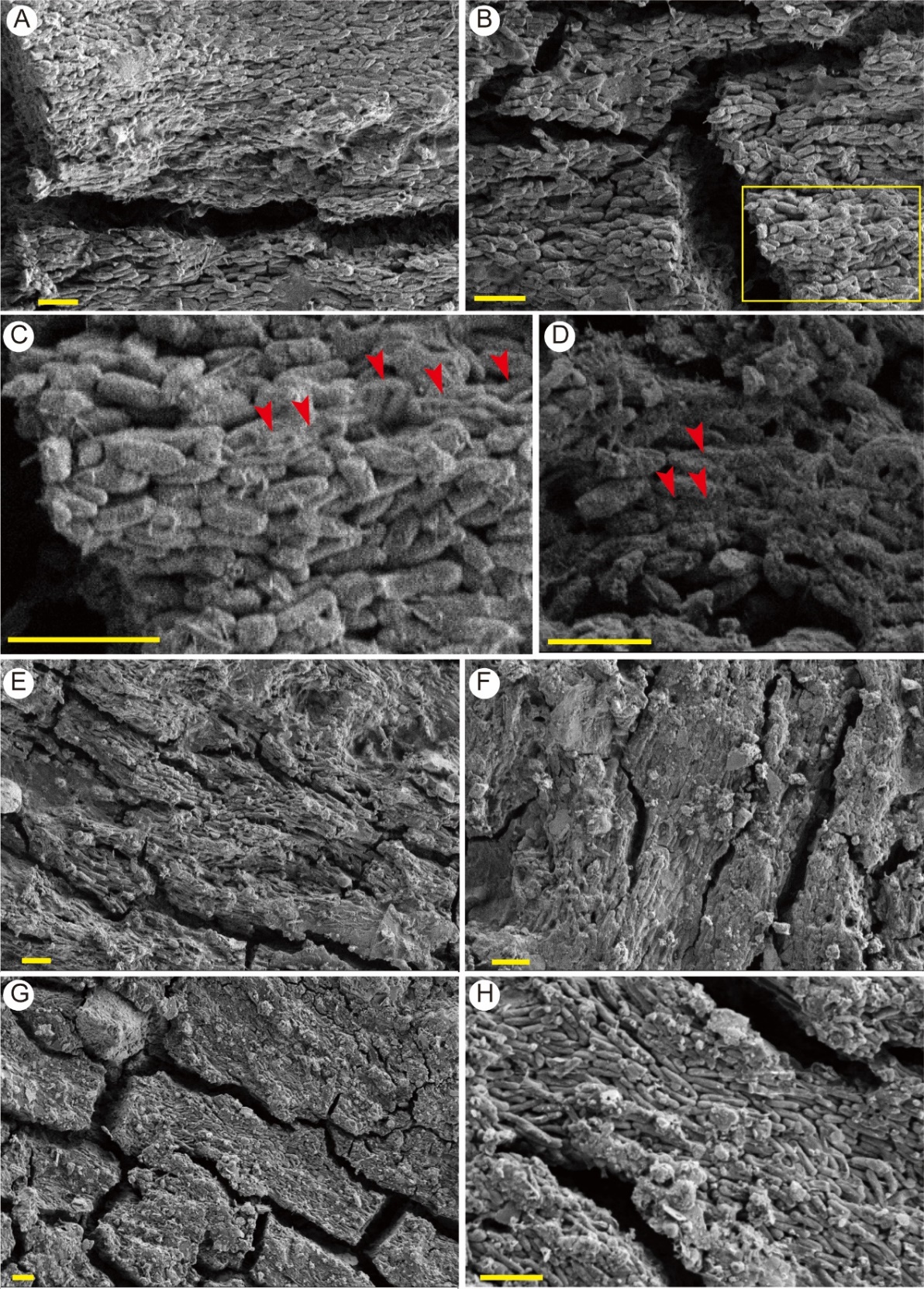


Fig. S1. SEM images showing the melanosomes of the feather samples collected from *Eoconfuciusornis* STM 7-144. A-D, SEM images of the sample 201; C, high-magnification image of the boxed area in B, with some broken melanosomes showing the hollows structures (arrowed); D, high-magnification image showing some fused melanosomes due to taphonomy (arrowed); E, SEM images of the sample 202; F, SEM images of the sample 203; G, SEM images of the sample 204. Scale bar is 2µm.


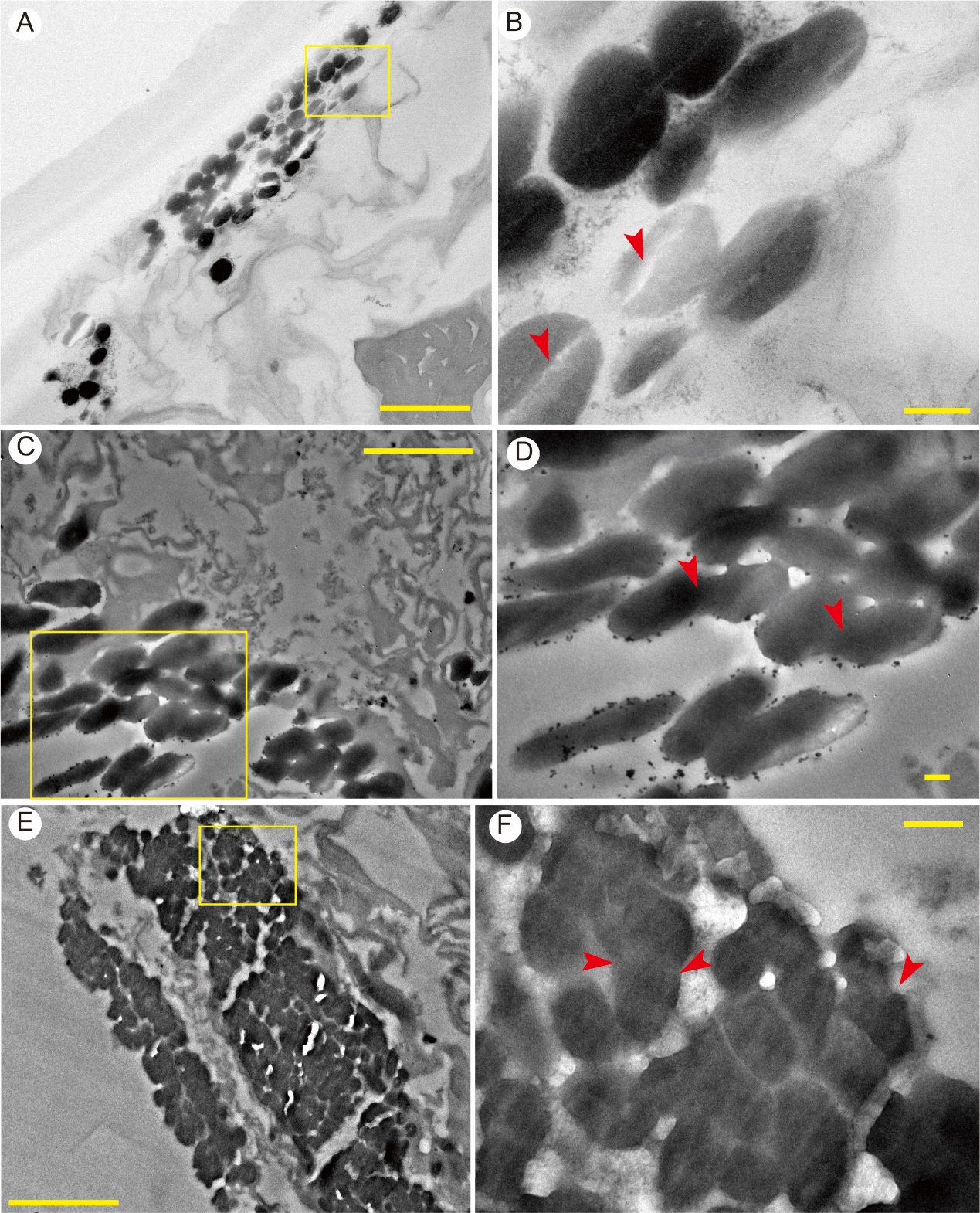


Fig. S2. TEM images showing the melanosomes of the feather samples 202 (A, B), 203 (C,D) and 204 (E, F) collected from *Eoconfuciusornis* STM 7-144, and none of them showing the hollow structures as observed in sample 201. A, C, E, low-magnification images, scale bar is 2µm; B, D, F, high-magnification images of the boxed areas in A, C, E respectively, scale bar is 200 nm, showing cracks and fusions of melanosomes (arrowed).


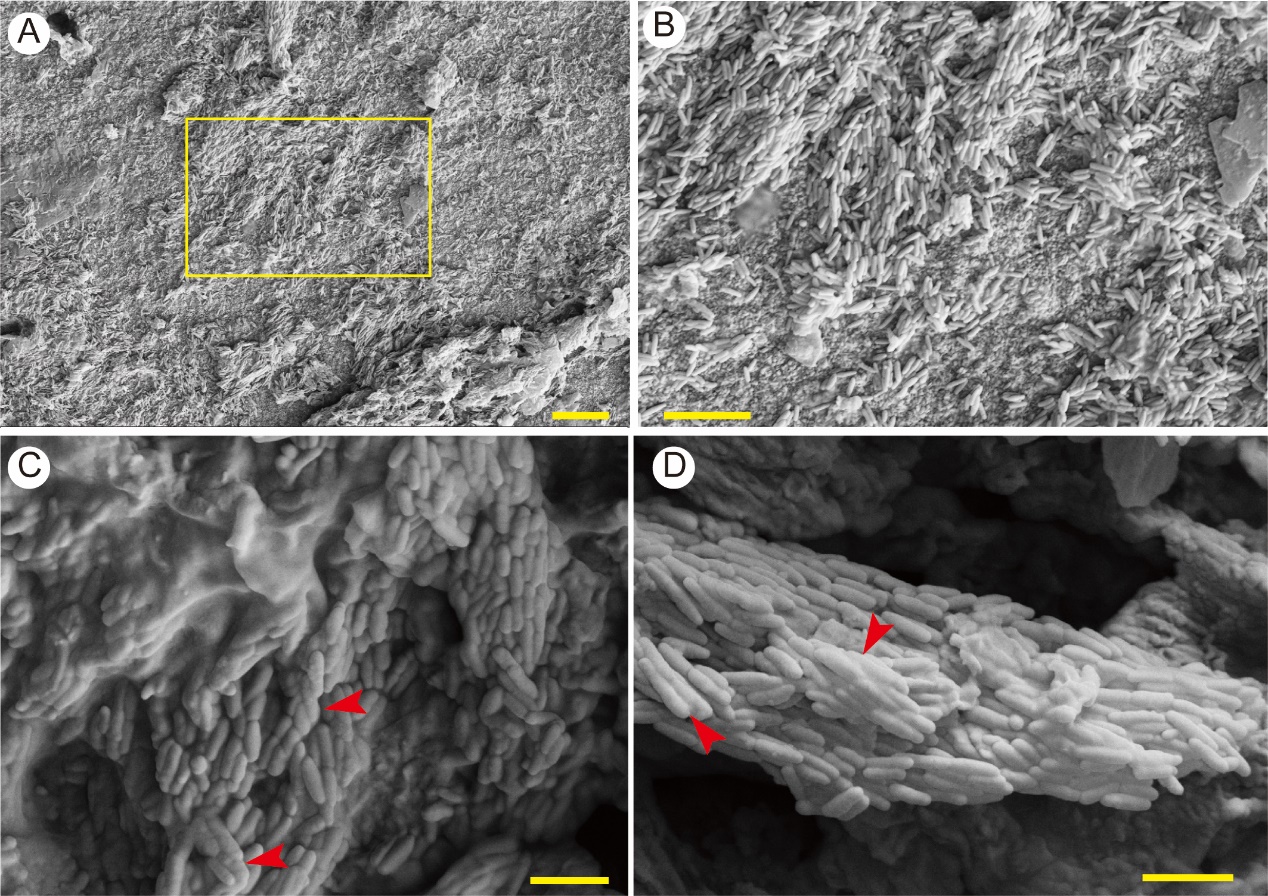


Fig. S3. SEM images of chicken feathers treated under condition 4 of the maturation experiment (1). A low-magnification image, scale bar is 10µm; B, high-magnification image of the boxed area in A, scale bar is 2µm; C, D, high-magnification images showing fused melanosomes (arrowed), scale bar is 1µm.


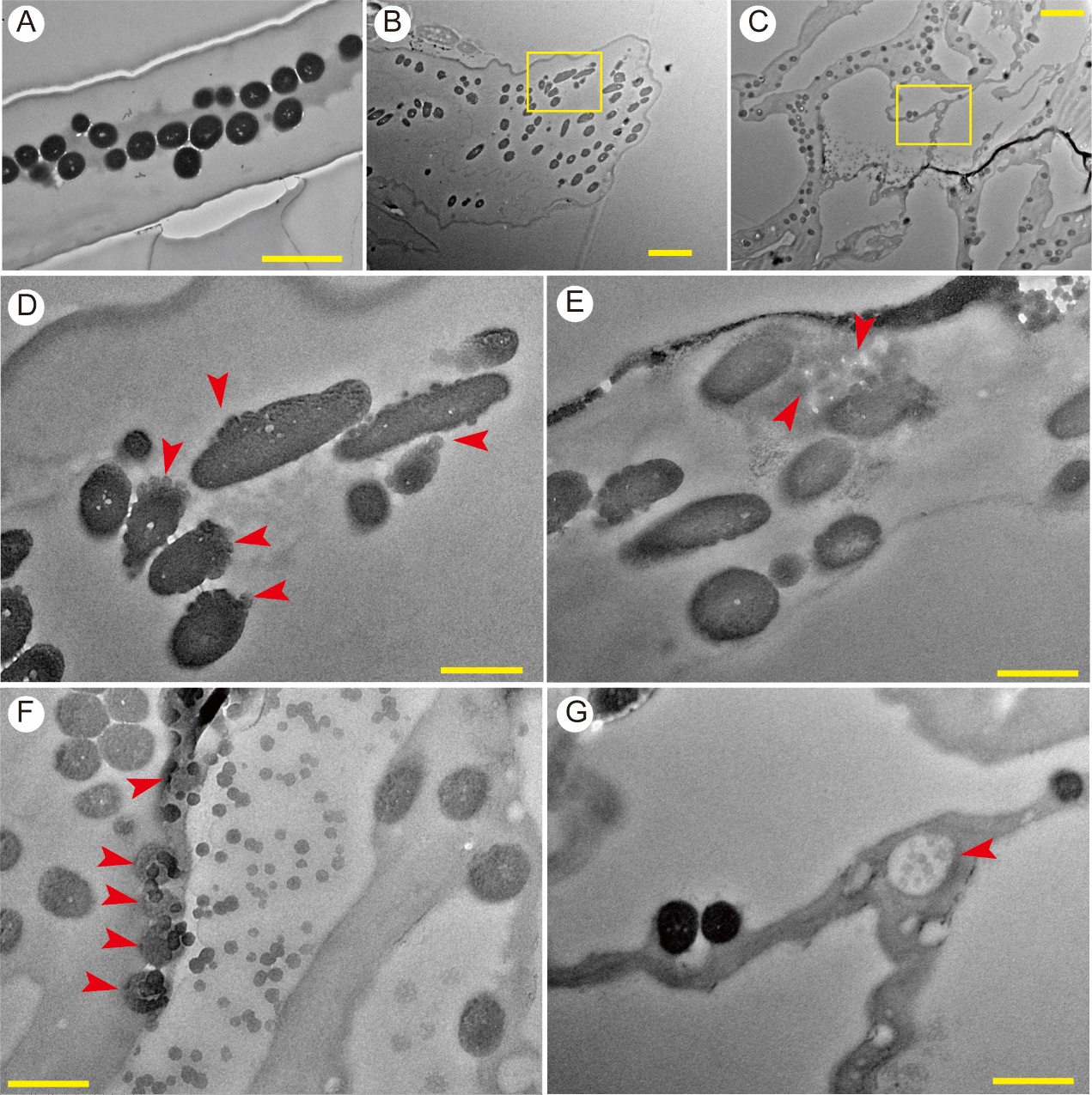


Fig. S4. TEM images of untreated and maturation experimentally treated chicken feathers showing the degradation of melanosomes. A, melanosomes from untreated feathers, scale bar is 1µm; B, melanosomes from feather treated under condition 2, scale bar is 2 µm; C, melanosomes from feather treated under condition 3, scale bar is 2 µm; D, high-magnification image of the boxed area in B, melanosomes are frayed at the edges, some of them with melanin granules scattered around the periphery (arrowed), scale bar is 500 nm; E, F, high-magnification image of melanosomes from treated feather under condition 3, the melanin granules flowed out, and the melanosomes with an uneven color under TEM (arrowed), scale bar is 500 nm; G, high-magnification image of the boxed are in C, a hollow with distinct margin when almost all melanin gone (arrowed), scale bar is 500 nm.

Supplementary Text 1

*Material and methods*

*Eoconfuciusornis* is the earliest branching stem avialan with a beak, found in the Huajiying Formation (~130.7 Ma), the oldest fossil-bearing stratum of the Jehol Biota [1]. One of the *Eoconfuciusornis* specimens STM 7-144 has been extensively sampled for melanosomes [1, 2],

The feather samples were embedded in resin following the protocol from Pan et al. (2016) [2], and then cut into 100 nm thick cross sections with a Leica UC7 ultramicrotome for analysis using TEM.

References:

1. Zheng X, O’Connor JK, Wang X *et al.* Exceptional preservation of soft tissue in a new specimen of *Eoconfuciusornis* and its biological implications. *Natl Sci Rev* 2017;**4**:441–52.

2. Pan Y, Zheng W, Moyer AE *et al.* Molecular evidence of keratin and melanosomes in feathers of the Early Cretaceous bird *Eoconfuciusornis*. *Proc Natl Acad Sci* 2016;**113**:E7900.

Supplementary Text 2

*Taphonomic alteration of melanosomes*

Experimental studies of taphonomic alterations can help to better interpret fossils, and maturation experiments simulating diagenesis is commonly applied in experimental taphonomy studies (e.g. 1-4). Here we examined the alteration of melanosomes during maturation experiments, and compared with the fossil melanosomes of *Eoconfuciusornis* (STM7-144). The flight feathers from a domestic chicken (*Gallus gallus*) were wrappled in aluminum foil and matured under 250 bars at four different conditions: 1) 100℃ for one hour; 2) temperature gradually increased from 100℃ to 150℃ over one hour and kept at 150℃ for one hour; 3) temperature gradually increased from 150℃ to 200℃ over one hour and kept at 200℃ for one hour; 4) temperature gradually increased from 200℃ to 250℃ over one hour and kept at 250℃ for one hour. The results from condition 1 to condition 4 showed the alteration process of feathers, and more details see Zhao et al. 2020 (1).

Here we showed that under condition 4, when the feather has turned into ashes and some yellowish liquid, numerous exposed melanosomes were observed (Fig. S3). At higher magnifications, some of the melanosomes were morphologically changed and becoming fused together (arrowed in Fig. S3 C and D). TEM observations (Fig. S4) provide additional details to SEM observations on the degradation process of the melanosomes as also described in Zhao et al. 2020 (1): first, melanosomes are frayed at the edges, some of them with melanin granules scattered around the periphery (arrowed in Fig. S4, D, E); then, the melanin granules flowed out, and the melanosomes with an uneven color under TEM (arrowed in Fig. S4 E, F); finally, almost all melanin are gone and left a hollow within the keratinous matrix (arrowed in Fig. S4 G). During this progress, when compaction happened, more fused melanosomes will be formed.

The maturation experiments showed that during degradation melanosomes may fuse together or leave hollows with indistinct margins after deep degradation. While the air holes in hollow melanosomes are circled by a uniform thickness of melanin layer, which is distinguishable from the hollows formed by degradation.

References

1. Zhao T, Hu J, Hu L, Pan Y. Experimental maturation of feathers: implications for interpretations of fossil feathers. *Palaios*, 2020; **35**: 67-76.
2. McNamara ME, Briggs DEG, Orr PJ, Field DJ, Wang Z. Experimental maturation of feathers: implications for reconstructions of fossil feather colour. *Biology Letters* 2013; **9**: doi: 10.1098/rsbl.2013.0184.
3. Moyer AE, Zheng W, Schweitzer MH. Keratin durabiligy has implications for the fossil record: results from a 10 year feather degradation experiment. *PLOS ONE* 2016; **11**: doi: 10.1371/journal.pone.0157699.
4. Saitta ET, Kaye TG, Vinther J. Sediment-encased maturation: a novel method for simulating diagenesis in organic fossil preservation. *Paleontology*, 2019; **62**: 135-150.
